# Prevalence and genomic characteristics of zoonotic gastro-intestinal pathogens and ESBL/pAmpC producing *Enterobacteriaceae* among Swedish corvid birds

**DOI:** 10.1080/20008686.2019.1701399

**Published:** 2019-12-12

**Authors:** Robert Söderlund, Hanna Skarin, Stefan Börjesson, Axel Sannö, Therese Jernberg, Anna Aspán, Erik O. Ågren, Ingrid Hansson

**Affiliations:** a Department of Microbiology, National Veterinary Institute, Uppsala, Sweden; b Division of Microbiology, Infection and Inflammation, Linköping University, Linköping, Sweden; c Department of Clinical Sciences, Swedish University of Agricultural Sciences, Uppsala, Sweden

**Keywords:** One health, zoonotic disease, campylobacter, wildlife, genomics, jackdaw

## Abstract

**Introduction**: Wild birds pose a potential threat to animal and human health by spreading infectious diseases. In the present study, we studied the occurrence of bacterial zoonotic pathogens as well as enterobacteria with transferrable antimicrobial resistance genes among Swedish corvids.

**Materials and methods**: Intestines from 66 jackdaws, crows, rooks and magpies from the vicinity of livestock farms at 14 locations in 7 counties were analysed by direct culture or PCR screening followed by culture. Isolates were investigated by whole-genome sequencing.

**Results and discussion**: *Campylobacter jejuni* were detected in 82% and *Yersinia* in 3% of the birds. ESBL-producing *E. coli* were found in one sample (2%) and carried *bla*_CTX-M-55_. No *Enterobacteriaceae* with transferable carbapenem resistance were identified. No *Salmonella* or *E. coli* O157:H7 were found, but PCR analysis for enterohaemorrhagic *E. coli* virulence genes revealed 35% positive samples for intimin, 9% for verotoxin 1 and 17% for verotoxin 2. *C. jejuni* isolates from corvids were compared to previously published isolates from Swedish sources by multi-locus sequence typing based on genome sequences. All corvid *C. jejuni* isolates formed a cluster, intermingled with human and chicken isolates. Our results indicate that *C. jejuni* is ubiquitous among Swedish corvid birds, with sporadic transmission to poultry and humans.

## GRAPHICAL ABSTRACT

**Figure:**
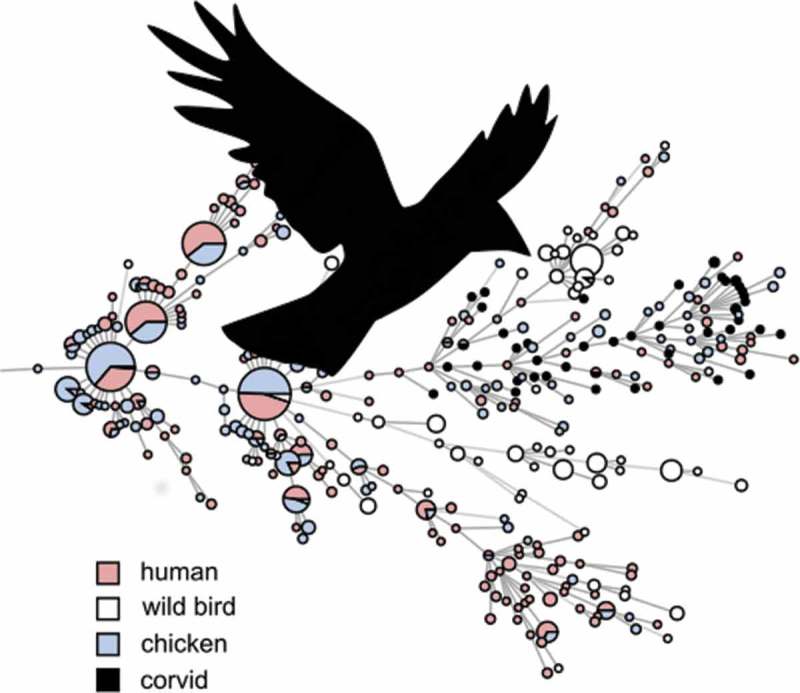

## Introduction

Zoonotic gastrointestinal diseases cause a significant burden of disease worldwide, with discomfort and a risk of disability or death for affected individuals as well as substantial costs for society [1]. The increasing trend of antibiotic resistant bacteria among food-producing animals is also a growing concern for human and animal health, particularly the occurrence of extended spectrum beta-lactamase (ESBL) and transferable ampC (pAmpC) producing Enterobacteriaceae. The four most commonly reported zoonoses in the EU are infection by *Campylobacter, Salmonella, Yersinia*, and shiga/verotoxin-producing *Escherichia coli* (STEC/VTEC) [2]. All four of these are known to have primary reservoirs among food-producing animals like ruminants, pigs or poultry. Whereas foodborne transmission, environmental transmission and direct contact with animals are known routes of infection, most sporadic cases of the major zoonoses in Sweden cannot currently be traced to a source. The role of wildlife as vectors and secondary sources of infection is poorly investigated in this regard and has been suggested as a key knowledge gap for reducing the number of human cases [3]. Studies performed in recent years have shown a strong regionality in terms of genotype for endemic zoonotic pathogens like STEC/VTEC [4] and *Salmonella* [5] in Sweden. Given that local transmission appears to be the dominant mode of movement between farms for these pathogens, rather than e.g. long-range trade of live animals, the role of wildlife as a vector is interesting. In addition, several international studies have shown high occurrence ESBL-producing *Enterobacteriaceae* in wildlife, primarily in birds including corvids [6,7]. Wild birds pose a particular challenge for farm biosecurity and food safety as they roam over large areas and are difficult to keep out.

Eight species of corvids are resident in Sweden, of which the jackdaw (*Corvus monedula*), the hooded crow (*Corvus corone*, also known as *Corvus cornix*), the rook (*Corvus frugilegus*), the raven (Corvus corax) and the magpie (*Pica pica*) are omnivores with frequent interactions with humans and domestic animals. The remaining three species (Eurasian and Siberian jay, and spotted nutcracker) are more reclusive and have specialized feeding habits [8]. In the present text we will for convenience exclude the latter three species when referring to corvids. Corvid birds are known to forage in the countryside, e.g. eating crops and animal feed as well as finding food in refuse heaps and collecting insect larvae from animal faeces [9,10]. They also commonly seek out human habitations and scavenge for food in garbage, at outdoor restaurants and in recreational areas [10,11]. Jackdaws are known to assemble in flocks in cities during the evening and night to socialize and sleep [10]. These flocks frequently become a nuisance due to faecal contamination and noise but are notoriously difficult to get rid of [10]. Thus, corvids are strong candidates to play a role both in the transmission of zoonotic pathogens between food-producing animals and in causing sporadic cases of infection among humans that are difficult to trace to a source.

The aims of the present study were to investigate the occurrence of *Campylobacter, Salmonella, Yersinia* and the STEC/VTEC serotype O157:H7, as well as carbapenemase and extended spectrum cephalosporinase-producing *Enterobacteriaceae* (CPE and ESCE), among corvid birds in rural areas in southern Sweden, and to determine the relevance of any findings by genomic comparison to isolates previously recovered from humans or domestic animals.

## Materials and methods

### Collection and handling of samples

**Figure 1.**
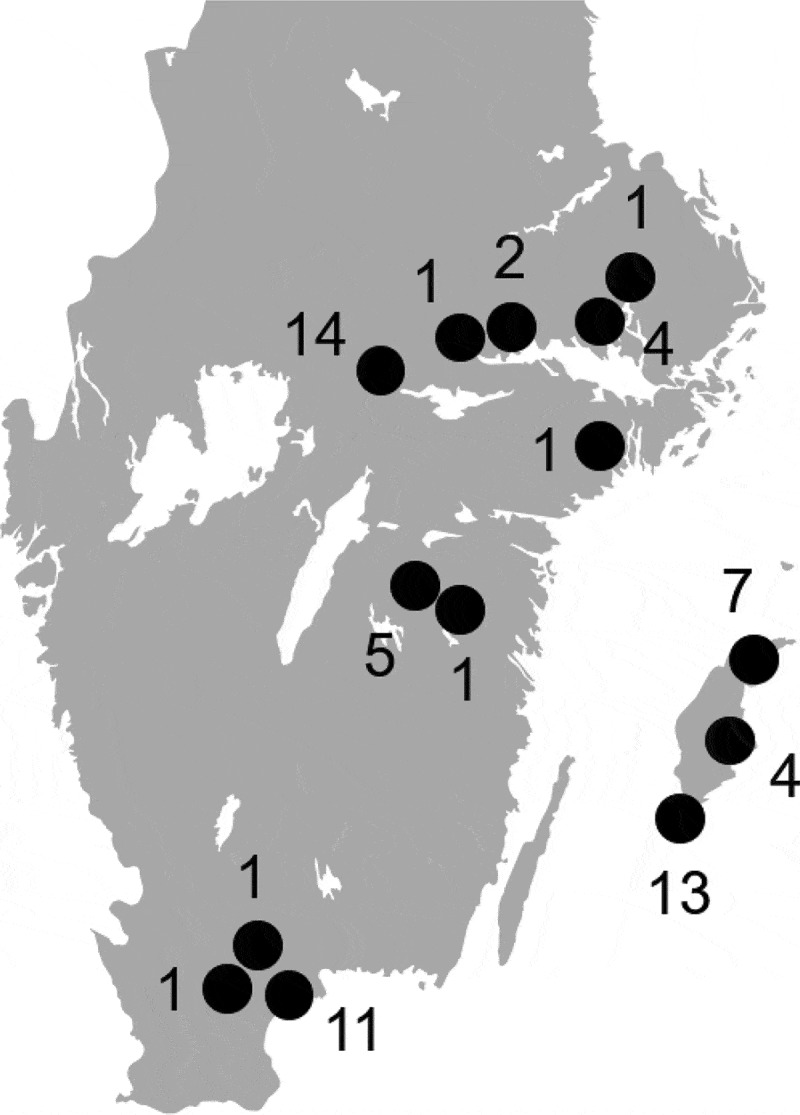
Approximate sampling locations (black dots) and the number of birds sampled at each location.

All birds were collected by Swedish hunters recruited via hunting networks and personal contacts. The submitted birds were culled for nuisance wildlife management in the Swedish countryside. All birds were collected near cattle, pig, poultry or sheep farms (< 3 km) in the southern part of Sweden where the density of domestic animal farms and the human population density is higher. A total of 66 birds, 43 jackdaws (*C. monedula*), 17 hooded crows (*C. corone*), 4 rooks (*C. frugilegus*), and 2 magpies (*P. pica*) were collected on 18 sampling occasions in March-December of 2017 at 14 locations (Figure 1) in 7 counties (Skåne, Gotland, Östergötland, Örebro, Västmanland, Stockholm and Uppsala). Hunters were instructed to submit whole birds by mail at ambient temperature. Two intestinal segments with content, each including one of the caeca, were excised from each submitted bird the same day they arrived at the laboratory, and analysis for VTEC/STEC O157:H7 and *Campylobacter* was initiated using one of the two segments within 24 hours of the excision. The other intestinal segment was frozen at −70°C in buffered peptone water (BPW) supplemented with 15% glycerol for later analysis for *Salmonella* spp., *Yersinia* spp., non-O157 VTEC/STEC, CPE and ESCE.

### PCR analysis and isolation of pathogens

The occurrence of *Campylobacter* spp. was analyzed according to ISO 10272 part 1 [12]. In brief, about 0.5 g (a loopful) of fresh caecal content from each sample was spread on modified charcoal-cefoperazone desoxycholate agar (mCCDA) plates (Oxoid), which were incubated at 41.5 ± 1°C for 44 ± 4 h in a microaerophilic atmosphere generated using the Mart BV Anoxomat system. Suspected *Campylobacter* colonies were cultured on blood agar and incubated at 41.5 ± 1°C for 44 ± 4 hours, and confirmation and species identification was performed using a Bruker Biotyper MALDI-TOF mass spectrometer. Ten samples were not analysed for *Campylobacter* due to staff shortages and the need for analysis of fresh material.

Analysis for VTEC/STEC O157:H7 was performed after *Campylobacter* analysis on the intestinal segment with content by pre-enrichment followed by immunomagnetic separation (IMS) as previously described [13].

Analysis for *Salmonella* spp. and *Yersinia* spp. was performed on frozen samples which were thawed and pre-enriched in BPW added to a ratio of 9:1 followed by overnight incubation at 28°C. The pre-enriched BPW was plated on brilliant green (BG), xylose lysine deoxycholate (XLD) and cefsulodin irgasan novobiocin (CIN) agar. Colonies with morphologies consistent with *Salmonella* were selected from the BG and XLD plates and suspended in 4 ml brain-heart infusion (BHI) liquid medium; in the absence of such colonies the BHI was inoculated from the primary streak of the plate. Separate BHI cultures for *Yersinia* were prepared in the same way from the CIN plates. From each BHI suspension 100 µL was then mixed before DNA was extracted using Bio-Rad InstaGene Matrix according to the manufacturer’s instructions. The remaining BHI suspensions were supplemented with 15% glycerol before storage in −80°C for subsequent analysis. Dual-labelled probe real-time PCR was performed for *Salmonella* [14], *Y. enterocolitica* [15] and *Y. pseudotuberculosis* [16] on the DNA extracts. Any sample with a threshold cycle (C_t_) < 35 was considered positive. Positive samples were re-plated from the frozen BHI on XLD/BG/CIN and colonies confirmed by the same real-time PCR, performed on colony lysates.

For detection of CPE and ESCE 0.1 ml of frozen samples was added to 9.9 ml fresh BPW and incubated overnight at 37°C, after which 10 μl of broth was transferred to CHROMagar, SuperCARBA and MacConkey agar supplemented with 1 mg/L cefotaxime. Based on phenotypic appearance, colonies of suspected *Enterobacteriaceae* were selected from each plate and streaked on horse blood agar. Confirmation of bacterial species was performed using a Bruker Biotyper MALDI-TOF MS. Identified isolates of *Escherichia coli, Klebsiella* spp., and *Enterobacter* spp. were subjected to WGS for confirmation as CPE or ESCE.

Parallel pre-enriched BPW samples were screened using dual-labelled probe real-time PCR targeting the primary VTEC/STEC virulence factors vero/shigatoxins 1 (*stx_1_*), 2 (*stx_2_*) [17] and intimin (*eae*) [18].

### Whole genome sequencing and characterization of isolates

Colony mass was collected from plates and used for DNA extraction with the Qiagen blood and tissue kit, automated on a Qiagen EZ1 Biorobot. Extracted DNA was used for Illumina Nextera XT library preparation according to the manufacturer’s instructions and sequenced to >50x coverage paired-end 2 × 250bp on an Illumina MiSeq instrument. Sequence data were uploaded to the European Nucleotide Archive (ebi.ac.uk/ena) and are publicly available under project accession number PRJEB34530. MLST for *Yersinia* [19] was performed using tools at EnteroBase [20], whereas *Campylobacter* and *E. coli* MLST was performed using the Center for genomic epidemiology (CGE) MLST tool [21]. New loci and sequence types found were submitted to the databases. Reference data for *C. jejuni* MLST was downloaded from pubMLST including all available profiles from isolates from humans (n = 457), chickens (n = 415) and wild birds (n = 446, of which 6 from corvids). Sequence-based *E. coli* serotyping was performed using the CGE SerotypeFinder tool [22]. *In silico* PCR was used to determine the presence of *Yersinia* virulence genes *ail, ystA, ystB, inv, yadA, virF*/*lcrF* [23] and *irp2* [24], on assemblies created using SPAdes 3.5.0 [25] run with the – careful flag. Resistance genes were detected using ARIBA and the ResFinder database (2019-03-29) [26].

### Statistical methods

Confidence intervals for prevalence estimates were calculated using the Agresti-Coull method [27] implemented in the *binom* package in R 3.3.1. Minimum spanning tree analysis was performed in BioNumerics 7.6 using all MLST markers as equivalent categorical variables.

## Results

**Figure 2.**
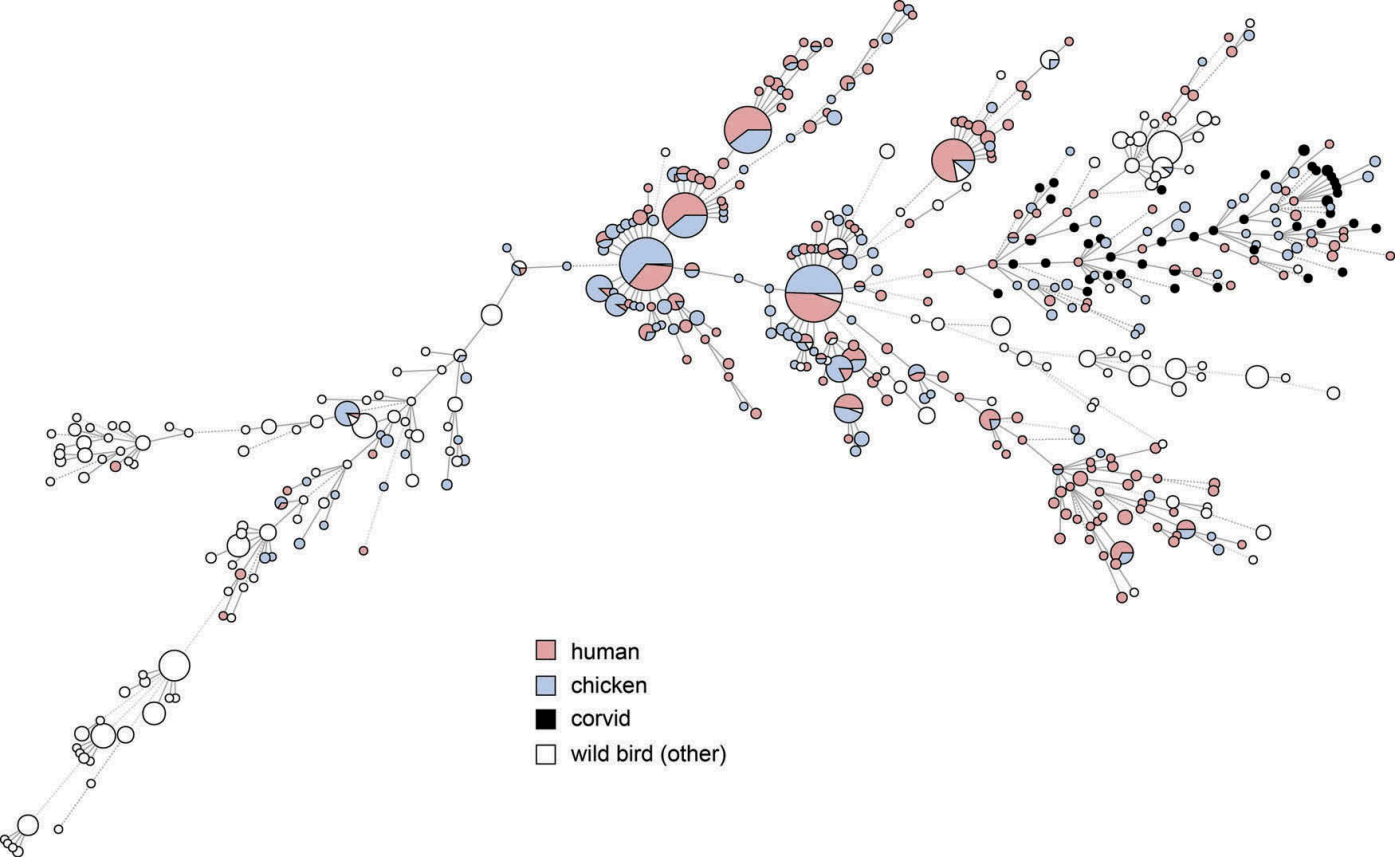
Minimum spanning tree of MLST data from *C. jejuni* isolated from human cases of gastroenteritis, chicken farms and wild birds including corvids in Sweden (n = 1357). Distances of 1–3 loci are shown as solid lines, 4 loci as dashed lines.

**Figure 3.**
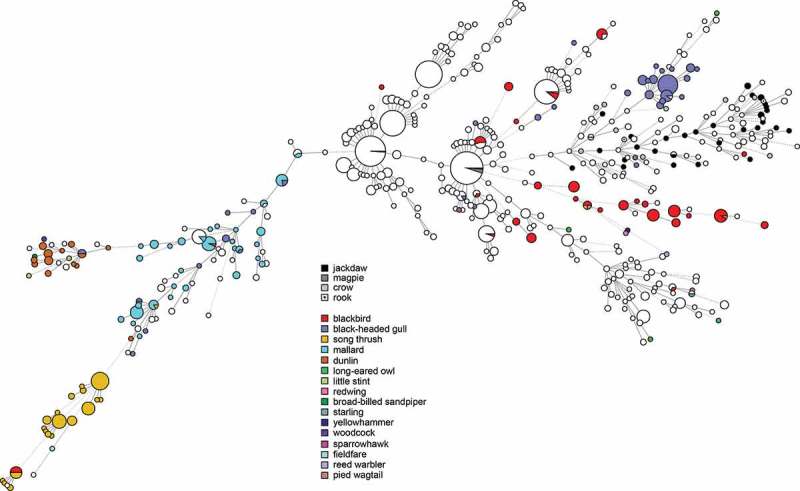
Minimum spanning tree of MLST data from *C. jejuni* isolated from human cases of gastroenteritis, chicken farms and wild birds including corvids in Sweden (n = 1357), showing species of wild birds for comparison with Figure 2.

*Campylobacter* were isolated from the caeca of 46/56 birds (82%, 95% CI 70–90%). *Campylobacter* was detected in samples from all locations and all bird species. All isolates were identified as *C. jejuni*. Five isolates were found after genome analysis to be contaminated by at least one additional *Campylobacter* strain, likely due to the carriage of multiple strains by the sampled birds, and one isolate was contaminated by *Enterobacter cloacae*. These were excluded from further genomic characterization. 38 unique MLST profiles were identified among 39 isolates successfully characterized by *in silico* MLST; one isolate from a magpie (SVAcorv_33) lacked complete alleles at two loci (*glyA, tkt*) despite being sequenced to high coverage (>100x) and could not be characterized. Although the diversity as determined by MLST was high, all corvid isolates formed a diffuse group together with several isolates from chickens and human cases (Figure 2). The corvid isolates did not cluster with profiles from other wild birds in Sweden except for a single isolate each from a blackbird (*Turdus merula*) and a black-headed gull (*Chroicocephalus ridibundus*) (Figure 3). Most isolates (31/40) carried an OXA type beta-lactamase gene, showing >99% similarity to *bla*_OXA-446_ or *bla*_OXA-449_, with 11 out of 31 of these isolates also positive for the *tet*O gene conferring tetracycline resistance (>99% match). One of the isolates with both *bla*_OXA-like_ and *tet*O also contained hits for the *cfr* (97% match to KX686749), *ant*6-Ia (94% match to AB247327) and aph(3ʹ)-III (93% match to M26832) genes. One isolate was also positive for the genes *tetO*, ant(6)-Ia (94% match to AB247327), aph(3ʹ)-III_(93% match to M26832), and the *cfr* (97% match to KX686749) and one isolate only carried *tet*O.

Two birds carried *Yersinia* spp. as determined by real-time PCR, one *Y. enterocolitica* and the other *Y. pseudotuberculosis* (in total 3.0%, 95% CI 0–11%). Only the *Y. enterocolitica* isolate was successfully isolated. Whole-genome sequencing results revealed the isolate to have the MLST profile 2-5-2-2-79-11-6 and the virulence gene profile *ail*+ *ystA*- ystB+ *inv- yadA- virF*/*lcrF- irp2-.*

Neither *E. coli* O157:H7 nor *Salmonella* spp. was detected in any sample in the study (0%, 95% CI 0–7%).

PCR analysis for key VTEC/STEC virulence genes revealed 35% positive samples for *eae*, 9% for *stx_1_* and 17% for *stx_2_*. 10/66 samples (15%) were positive for *eae* together with one or both *stx* subtypes.

Carbapenemase-producing *Enterobacteriaceae* (CPE) were not detected in any of the samples. Four suspected ESBL-producing ESCE were found in four separate samples; three *E. coli* and one *E. cloacae*. Two of the *Escherichia coli* and the *Enterobacter cloacae* was found by whole-genome sequencing to have no ESBL or pAmpC genes. Both the *E. coli* isolates had point mutations in the *ampC* promoter region giving a likely explanation to the observed phenotype and the *E. cloacae carried a bla*_ACT_ which likely is chromosomally encoded. A single isolate of *E. coli* O80:H19 ST-165 was found to carry the ESBL gene variant *bla*_CTX-M-55_. This isolate also carried the resistance genes *arr*2, *acc*(3)-IId, *aad*22, *aph*(3ʹ)-Ia, *aph*(3ʹ’)-Id, *aph*(6)-Id, *cmlA1, dfr14, floR*, InuF, *mefB, mdfA, mphA, qnrS1, sul3*, and *tetA*.

## Discussion

*Campylobacter jejuni* is clearly ubiquitous among corvids in rural parts of southern Sweden, as the bacteria were found in 82% of samples analysed in the present study. Although *Campylobacter* can be found in samples from a wide variety of birds, the bacteria have been described as particularly prevalent among scavengers and omnivores such as corvids [28], consistent with this observation. A high prevalence of *Campylobacter jejuni* among crows (39–73%) has been reported in previous studies performed on multiple continents [28–30]. Other studies have observed *C. jejuni* in both jackdaws and magpies [28,31].

Previous molecular source attribution efforts based on MLST have indicated a high degree of host bias among lineages of *C. jejuni* in wildlife; in contrast certain ‘farm genotypes’ are frequently shared between chickens and cattle [32]. Genotypes associated with farm animals are occasionally found in wild birds, but genotypes associated with wild birds have been reported to be less likely to be found in domestic animals [31]. As evident in Figure 3, the same phenomenon appears to be true for most wild bird genera in Sweden. In contrast, our results show that the cluster associated with corvids in the present study is relatively common among both human and chicken isolates in Sweden (Figure 2). We also note that several STs found in the present study have been isolated from human cases of gastroenteritis in the past in Sweden (ST-6591, ST-6609), Denmark (ST-8862), and the UK (ST-6591), as reported in PubMLST, demonstrating their zoonotic potential. Although based on a small convenience sample, this indicates that the tendency of corvids to seek out humans and domestic animals can result in transmission of *Campylobacter* more frequently compared to other wild birds. Corvids have been known to cause outbreaks and sporadic cases by contaminating the environment and food; as an example, magpies and jackdaws pecking the caps of milk bottles caused a *Campylobacter* outbreak in England in 1990 [11]. Later studies in the UK found that 2.1–3.5% of human campylobacteriosis cases in Oxfordshire could be attributed to wild birds [33], and that wild birds were especially common as a source of infection for children in rural parts of Scotland during the summer [34]. The majority of human cases of campylobacteriosis are believed to be of foodborne origin, with poultry considered to be the main source of domestic cases in Sweden [35], as well as in the EU as a whole [36]. Interestingly, the corvid cluster occurs among chickens and humans in the presented data, but the opposite is not true; the major STs that cause campylobacteriosis among humans and chickens in Sweden do not occur among corvids. This could indicate a largely one-way transmission of infection, perhaps because most corvids appear to be colonized by *C. jejuni* and may carry genotypes adapted to corvid hosts. The high number of isolates from corvids with the tetracycline resistance gene *tetO* is interesting and raises questions on the origins of the corvid strains. The gene detected in the present study is to the author’s knowledge the only gene conferring tetracycline resistance described in *Campylobacter*. A recent study conducted in California observed that the *tet*O gene primarily was linked to isolates from American crows (*Corvus brachyrhynchos*) [37]. Together with the present observations, this indicates a possible link between corvid-adapted lineages and the *tet*O gene. However, *tet*O genes have been found to also occur in *C. jejuni* from a variety of other sources, and to frequently be carried on a conjugative plasmid encoding a type IV secretion system and several other genes of unknown function [38]. It is therefore possible that this plasmid contributes to the fitness of *C. jejuni* strains even in the absence of selection from tetracycline. The occurrence of tetracycline resistance in Swedish clinical cases of *Campylobacter* in humans has been around 30% during recent years [39]. In contrast the occurrence of tetracycline resistance among Swedish poultry isolates has varied dramatically during the same period, despite no use of tetracyclines in poultry production [39], illustrating that resistant clones can expand in the absence of selective pressure from antibiotics.

The only isolate of *Yersinia enterocolitica* was positive for the gene encoding the Ail outer membrane protein found in pathogenic *Yersinia*, and the heat-stable enterotoxin Y-STb, but negative for all other virulence markers investigated including markers for the pYV virulence plasmid and the chromosomal high pathogenicity island. However, strains with such reduced sets of known virulence genes have been observed to frequently cause gastroenteritis in several countries and may to some extent rely on other, uncharacterized virulence factors [40].

In the present study only a single isolate of *Enterobacteriaceae* carrying an ESBL gene was found in a Swedish corvid, an *E. coli* carrying *bla*_CXT-M-55_. In a previous study on rooks in Europe the occurrence of *Enterobacteriaceae* carrying an ESBL genes varied from 0% in Spain to 25% in the Czech Republic [7]. The authors of that study did however highlight that the large difference in occurrence more likely reflected environmental contamination at the specific roosting sites rather than the occurrence in the countries investigated. Worth noticing is in that study *bla*_CXT-M-55_ was identified in *E. coli* from northern Germany, but in ST10 isolates and not in ST165 as in the current study. *E. coli* belonging to ST58 and ST155 with *bla*_CXT-M-55_ has previously been isolated from gulls in Sweden with 3 out of 29 ESBL producing *E. coli* carrying this gene variant [41]. *E. coli* with *bla*_CXT-M-55_ occasionally occurs among humans in Sweden and has been found in low proportion of pigs slaughtered in 2015 and 2017 [42]. As the investigated corvids were collected in the southern part of Sweden where the population density and density of animal farms is comparatively high it is conceivable that the positive bird acquired the strain from the environment with humans or pigs as the ultimate source.

No *E. coli* belonging to the serogroup O157:H7, which is the most common cause of human VTEC/STEC cases in Sweden, was found in this study. This is consistent with the notion of ruminants as the primary reservoir of VTEC O157:H7, with corvids perhaps serving as occasional passive vectors of transmission between animals or from animals to humans. However, we note a surprisingly high prevalence of verotoxin genes as determined by PCR, with a total of 15% of samples positive for intimin together with one or both of the toxin types, suggesting the presence of possible VTEC. Investigating the role for wild birds in the transmission of other, rarer variants of human pathogenic VTEC could thus be of interest.

Swedish wildlife including birds such as passerines have been found to carry *Salmonella* and spread infection to humans [43]. In the present study, *Salmonella* was not detected in any of the investigated corvids. *Salmonella enterica* rarely occurs among Swedish food-producing animals, with the exception of *Salmonella* Dublin which is endemic in certain areas in the southern part of the country [5]. Transmission from domestic animals to wild birds is therefore likely to be minimal.

The samples analysed in the presented study were collected at 14 locations during a single season giving a reasonably good coverage of southern and south-eastern Sweden. However, for practical reasons the number of birds was limited, and the collection performed by volunteers in a non-systematic fashion. Therefore, the results should be interpreted with some caution, which is also evident in the wide confidence intervals of the prevalence estimates produced. In particular, the study design was not optimal for recovery of rare pathogens or passive carriership as could perhaps be expected e.g. for VTEC and *Salmonella*. As endemic clones of these pathogens tend to be geographically clustered in Sweden [4,5], targeted sampling of corvids in such hot-spots would perhaps show a different picture. In contrast, *Campylobacter jejuni* appears to be ubiquitous among Swedish corvids. Future molecular source attribution studies e.g. using core-genome MLST would likely benefit from including the presented genome data from corvids as a possible source. In broader terms, the influence of seasonality and biogeography on transmission of zoonoses in rural and peri-urban environments should be a focus area for future studies aimed at better explaining sporadic cases of human illness as well as farm-to-farm transmission.

## Data Availability

Data from high-throughput sequencing of the recovered bacterial isolates have been deposited in ENA (https://ebi.ac.uk/ena) under the accession number PRJEB34530. MLST profiles have been deposited in the pubMLST database (https://pubmlst.org) with isolate identities SVAcorv1-SVAcorv32, SVAcorv34-SVAcorv40.
